# One Year Outcomes and Stability of a Novel Scleral Anchored Intraocular Lens

**DOI:** 10.1155/2021/3838456

**Published:** 2021-08-26

**Authors:** Edoardo Abed, Matteo Forlini, Edlira Bendo, Aurelio Imburgia, Alessandro Mularoni, Corrado Gizzi, Giuseppe Santino, Daniele Corazza, Giacomo Costa

**Affiliations:** ^1^Department of Ophthalmology, Morgagni Pierantoni Hospital, Forlì, Italy; ^2^Department of Ophthalmology, San Marino State Hospital, Cailungo, San Marino

## Abstract

**Purpose:**

To assess one year results and stability of the implantation of a scleral anchored intraocular lens (IOL).

**Design:**

Interventional prospective case series.

**Methods:**

Sixty eyes of 60 patients affected by either aphakia or IOL dislocation were included in this study. Patients underwent vitrectomy, scleral fixation of the IOL, and, if present, dislocated IOL removal. Patients were evaluated preoperatively and at 1, 3, 6, and 12 months after surgery by best-corrected distance visual acuity (BCVA) assessment, intraocular pressure (IOP) measurement, corneal specular microscopy, and optical coherence tomography (OCT) of both the macula and anterior segment.

**Results:**

At twelve months, mean BCVA significantly improved (*p* < 0.0001), and none of the patients experienced a decrease of visual acuity. A 10% decrease of endothelial cell count occurred after surgery. Cystoid macular edema occurred in three patients (5%). A transient increase of intraocular pressure was noted in 7 cases (12%). At one month, horizontal and vertical IOL tilt was 1.04 ± 0.87 and 0.74 ± 0.71 degrees, respectively, and did not significantly change in the follow-up (*p* > 0.05). None of the patients had decentration or dislocation of scleral-fixated IOL during the follow-up.

**Conclusion:**

Implantations of scleral plug fixated IOL provide good visual results, low complication rate, and excellent stability of the lens until one-year follow-up.

## 1. Introduction

Intraocular lens implantation (IOL) in the capsular bag represents the gold standard in cataract surgery and provides excellent anatomical and functional outcomes [1]. However, lesions of the capsular bag including zonular dehiscence, posterior capsule rupture (PCR), and capsular bag luxation may occur due to cataract surgery complications [2], ocular trauma [3], high myopia [4], or pseudoexfoliation syndrome [5]. In these cases, capsular support is inadequate to allow standard in bag IOL implantation, and other surgical approaches should be adopted including anterior chamber IOL (ACIOL) implantation [6, 7], iris-fixated IOL [8, 9], and scleral-fixated IOL (SFIOL) [10, 11].

ACIOL and iris claw IOL are easy and fast to implant but might be associated with persistent inflammation, cystoid macular edema, and progressive endothelial cell loss with corneal decompensation [12, 13]. Various techniques have been proposed for scleral fixation of IOL. Classically, a rigid PMMA IOL is fixated to the sclera with prolene sutures. However, this procedure requires a large corneal incision, long operating times, and might be associated with late IOL dislocation due to loosening of sutures or erosion [10]. Furthermore, sutured scleral IOL is associated with significant optic tilt in over 50% of cases [14]. In the last few years, other techniques have been proposed for sutureless scleral fixation of a three-piece IOL with either fibrin glue [15–17] or by tucking the haptics into scleral tunnels [18, 19] or pockets [20]. Scleral gluing fixation technique has also been successfully combined with iris repair surgery [21]. Recently, a novel specially designed IOL with scleral plugs (Carlevale IOL) has been introduced as an option to correct aphakia without residual capsular support with good short-term outcomes [22] and low degree of decentration and tilt [23]. However, the long-term outcomes and stability of this implant have still not been explored.

The aim of this study is to evaluate the one-year outcomes and stability of the implantation of the scleral tucking Carlevale IOL.

## 2. Materials and Methods

### 2.1. Patients and Examination Protocol

**Figure 1:**
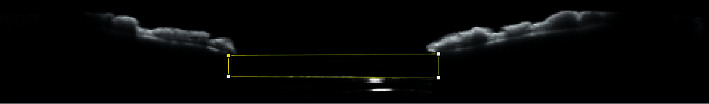
AS-OCT of a patient one month after FILSSF IOL implantation. Yellow boundary lines are drawn to calculate the angle between the implant and iris plane.

Sixty eyes of 60 patients who underwent SFIOL implantation between 1 November 2017 and 30 November 2019 at the Ophthalmology Department of Morgagni-Pierantoni Hospital and of San Marino State Hospital were enrolled in this prospective case series. The study adhered to the tenets of the Declaration of Helsinki and was approved by the Institutional Ethics Committee. A written informed consent was obtained from all patients. The trial was registered at ISRCTN (trial number: ISRCTN10015880). Inclusion criteria were postoperative or posttraumatic aphakia or late dislocation of IOL and/or capsular bag due to pseudoexfoliation syndrome (PEX). Patients with corneal opacities, visually significant macular diseases, retinal detachment, optic disk atrophy, advanced glaucoma, and any other ocular condition that was likely to compromise the functional outcome were excluded from the study. All patients underwent a complete ophthalmological examination including best-corrected visual acuity (BCVA) assessment, intraocular pressure (IOP) measurement with noncontact tonometry, slit-lamp biomicroscopy, and indirect ophthalmoscopy before surgery, seven days postoperatively, and at 1, 3, 6, and 12 months after surgery. Spectral-domain optical coherence tomography (SD-OCT) (Heidelberg Spectralis, Heidelberg, Germany) scans of the macular region were acquired at 1, 6, and 12 months postoperatively to assess the presence of cystoid macular edema. Specular microscopy (SP-1P; Topcon, Japan) was done at baseline and at 1, 6, and 12 months follow-up to assess postoperative endothelial cells loss. Anterior segment OCT (AS-OCT) (Heidelberg Spectralis, Heidelberg, Germany) was performed at 1 and 12 months after surgery to assess IOL tilt. A horizontal and a vertical 12 mm scans centered at the pupil were performed. Images were then exported in TIFF format and processed with ImageJ software. IOL tilting in the horizontal and vertical axis was assessed by measuring the angle between the IOL optic and the posterior iris surface plane (Figure 1). A horizontal or vertical tilt exceeding 5° was considered significant.

### 2.2. Surgical Procedure

**Figure 2:**
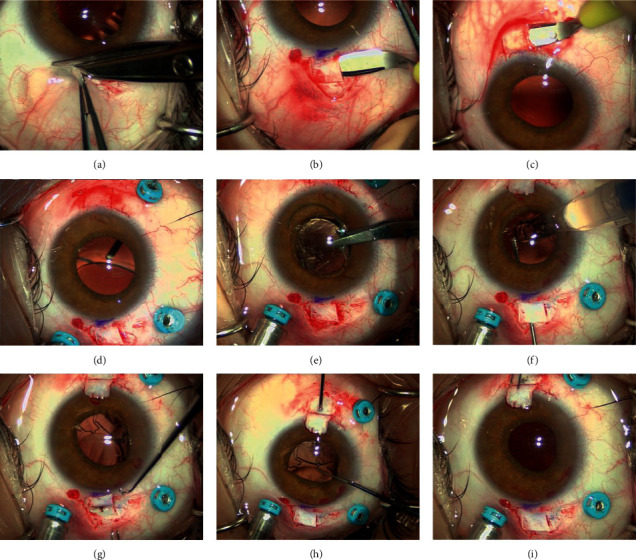
FILSSF IOL implant procedure: conjunctival peritomy (a), scleral flaps creation (b, c), vitrectomy (d), dilocated IOL removal (e), haptics grasping (f, h), and externalization (g, i).

Surgical steps of FILSSF IOL scleral fixation are summarized in Figure 2. Operations were carried out by three vitreoretinal surgeons (A.E., C.G., and M.F.) under peribulbar anesthesia. First, localized conjunctival limbal peritomy at nasal and temporal side and coagulation of bleeding vessel by bipolar cautery application were performed. Two limbal-based 3 × 3 mm scleral flaps of about one half of scleral thickness are then made in the nasal and temporal sides exactly 180° apart. Subsequently, a standard three-port, transconjunctival 23 or 25 gauge pars plana vitrectomy (PPV) (Constellation Vision System, Alcon Laboratories Inc., Fort Worth, Tex., USA) was performed. Posterior vitreous detachment was induced, and careful inspection of the periphery with scleral depression was made to detect retinal breaks. A noncontact wide-angle viewing system (BIOM, Oculus Inc., Wetzlar, Germany) was used during PPV for visualization. Two 23-gauge sclerotomies were then performed under the scleral flaps at 1.5 mm from the limbus by using the trocars. In case of 25-gauge vitrectomy, the sclerotomies were slightly enlarged. The following surgical steps slightly differed depending on the presence of an IOL dislocated in the vitreous chamber. In case of dislocation of a previously implanted IOL, the implant was first luxated in the anterior chamber with the cutter in aspiration-only mode or with vitreous serrated forceps and then removed through a 5.4 mm corneal incision performed in the superotemporal cornea. Conversely, in the absence of a dislocated IOL, a 2.2 mm keratotomy is framed in the temporal clear cornea for IOL introduction. Before IOL implantation, the anterior chamber is filled with ophthalmic viscosurgical device (OVD) (Viscotech, SIFI, Italy), and a paracentesis is performed in the nasal cornea. While the IOL is slowly introduced and unfolded in the anterior chamber with the injector, a 25-gauge vitreous serrated or end-gripping forceps are inserted through the nasal sclerotomy and used to grasp the center of the anchor-shaped tip of the leading IOL haptic. The haptic is then carefully pulled when the IOL is completely unfolded and externalized from the nasal sclerotomy.

Thereafter, the 25-gauge forceps are introduced from the temporal sclerotomy to grasp and externalize the IOL trailing haptic tip. In order to approach the trailing haptic to the pupil and allow its grasp with the forceps, a Sinskey hook is used from the nasal paracentesis.

At this point, the IOL is already centered and fixed without the need of further intrascleral tucking or suturing. If IOL vertical tilt is noticed, it may be easily corrected by gently rotating the anchor tips outside the sclerotomies. Scleral flaps and the conjunctiva are then sealed with Vicryl 7.0 suture. Trocar sclerotomies were carefully massaged and left unsutured unless leakage was observed. Corneal incision is sutured with Nylon 10.0 sutures only if a 5.4 mm tunnel was performed. If a 2.2 mm tunnel was performed, the incision is closed by wound hydration.

### 2.3. Statistical Analysis

Continuous variables were described as mean ± standard deviation and categorical variables as percentages. Pre- and postoperative values of BCVA, IOP, endothelial cell count, and the degree of IOL tilting at each follow-up were compared. For this purpose, analysis of variance for repeated measures and Sidak post hoc test were performed in order to avoid family-wise errors for multiple comparisons. For statistical purposes, Snellen BCVA was converted into logarithm of the minimum angle of resolution (logMAR). For all analyses, a *p* value <0.05 was considered statistically significant.

## 3. Results

**Figure 3:**
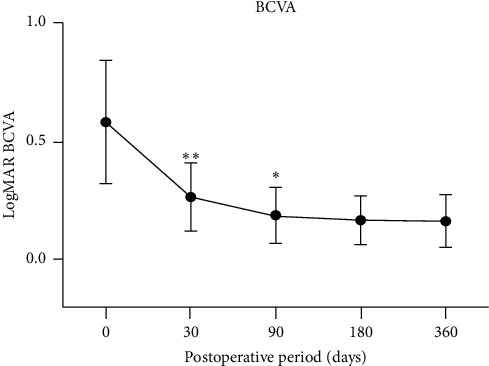
Graph showing the progressive postoperative improvement of BCVA. Visual acuity largely increased at 1 month, further improved at 3 months, and remained stable until the end of follow-up.

Sixty eyes of 60 patients met the inclusion criteria and were enrolled in the study. Forty-one patients (68%) were females and 19 were males (32%). The mean age was 79.3 ± 8.8 years. Preoperatively, thirty-three patients (55%) had late dislocation of a posterior chamber IOL which was due to progressive zonular laxity related to PEX in twenty-three cases (70%), trauma in three cases (9%), and high myopia in two cases (6%). In 20 patients (42%), aphakia was related to previous complicated cataract surgery. In three cases (5%), grasping the IOL with the end gripping forceps leads to haptic break. In such cases, the IOL was explanted trough the corneal incision and replaced with a FILSSF IOL with the same power. Three patients (5%) had intraoperative vitreous hemorrhage that spontaneously resolved at 1-month follow-up. No other intraoperative complications such as retinal breaks or detachment were noticed. BCVA was 0.58 ± 0.26 at baseline, increased to 0.26 ± 0.14 at 1 month (*p* < 0.001), and further improved to 0.19 ± 0.12 (*p* < 0.01) at three months after surgery. Afterwards, BCVA remained stable reaching 0.17 ± 0.11 at final follow-up (Figure 3). None of the patients experienced a decrease of BCVA after surgery.

Preoperatively, mean intraocular pressure (IOP) was 17.8 ± 6.7 mmHg and 10 patients (16%) had ocular hypertension (IOP > 21 mmHg) associated with PEX in 6 cases (60%) and with vitreous prolapse in the anterior chamber in the remaining four eyes (40%).

A postoperative increase of IOP, probably related to retained OVD, occurred in 5 patients (8%) and was successfully managed with topic hypotensive drugs. Persistent elevated IOP rise was noted in 2 cases (3%) who were already affected by pseudoexfoliative glaucoma.

At twelve-month follow-up, mean IOP significantly decreased to 14.2 ± 3.6 mmHg (*p*=0.01).

**Figure 4:**
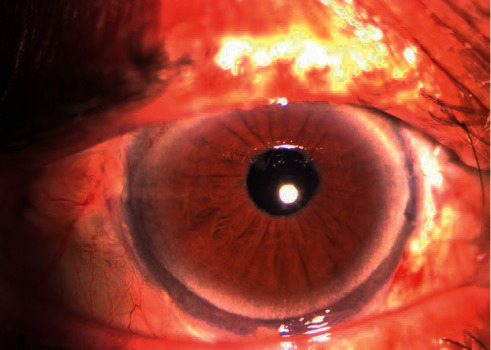
Slit lamp examination of a patient 1 month after surgery showing clear cornea, quiet anterior chamber, and perfect SSF IOL centration.

Before surgery, endothelial cell count was 1615 ± 502 cells/mm^2^ and dropped to 1481 ± 471 cells/mm^2^ one month after surgery. The difference reached statistical significance (*p* < 0.001). The density of endothelial cells further decreased to 1451 ± 457 cells/mm^2^ at final follow-up. Cornea was clear in all patients at one month after surgery (Figure 4), and none of the patients developed corneal edema or decompensation during the one-year follow-up.

**Figure 5:**
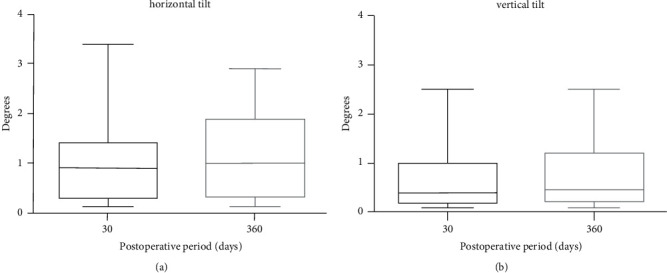
Box plot showing the stability of horizontal (a) and vertical (b) tilt of FILSSF IOL at 1 and 12 months after surgery.

The amount of horizontal and vertical SSF IOL tilt is summarized in Figures 5(a) and 5(b), respectively.

Horizontal IOL tilt was 1.04 ± 0.87° 30 days after surgery (range 0.1–3.4°) and remained unchanged at one-year follow-up (1.11 ± 0.86° *p*=0.20). Vertical tilt was 0.74 ± 0.71° one month postoperatively and 0.79 ± 0.65° at final examination (*p*=0.47). None of the patients had postoperative IOL dislocation, decentration, or significant vertical or horizontal tilt.

Postoperative retinal breaks and/or detachment were not observed in any of the patients included. Cystoid macular edema developed in three eyes (5%) at one-month follow-up and was successfully managed with topical and oral indomethacin in two cases and with dexamethasone implant in the remaining patient.

## 4. Discussion

Intraocular implantation in eyes with deficient capsular support is a therapeutic challenge for cataract surgeons, and multiple approaches have been proposed to manage these complicated cases. Placement of ACIOL or iris-fixated IOL is an easy and fast procedure but is associated with a large number of complications related to angle and iris tissue stimulation including endothelial cell loss, corneal decompensation, pigment dispersion, hyphema, secondary glaucoma, anterior uveitis, and cystoid macular edema [7–9]. To reduce the incidence of postoperative complications, particularly endothelial cell loss, retropupillary placement of iris-claw IOL has been proposed. However, a comparative case series by Toro et al. [24] did not show a significant difference between anterior and posterior iris claw IOL in terms of endothelial cells count. Intraocular lens fixation to the scleral wall provides a more physiological location of the implant, which lies near the ciliary body avoiding trauma and stimulation of the uveal tissue and thereby reducing inflammation-related ocular complications. The classical technique consists of rigid PMMA IOL suturing to the scleral wall with nonabsorbable prolene sutures. However, this technique requires a large corneal incision to introduce the IOL. Furthermore, an asymmetrical tension of prolene sutures may lead to significant IOL decentration and tilt which may cause significant astigmatism that may reduce visual recovery. In addition, erosion of progressive sutures may cause increasing IOL tilt and decentration or late dislocation of the IOL in the vitreous cavity.

In this study, we report the one-year functional and anatomical outcomes of the implant of FILSSF Carlevale IOL, a recently developed IOL specially designed for scleral sutureless fixation.

In three cases, intraoperative vitreous hemorrhage occurred but spontaneously resolved at one-month follow-up. No other major intraoperative complications were observed. In three cases, IOL leading to haptic break occurred after grasping it with end-gripping ILM forceps. This complication is related to the softness of IOL hydrophilic material and to the sharpness of the tip of ILM-forceps. For this reason, to reduce the risk of haptic break, we suggest using vitreous serrated forceps to manipulate the IOL.

Similar to glueing [14] or flanged intrascleral fixation [17] of three-piece IOL, FILSSF implantation requires a small corneal incision that can be left unsutured allowing low postoperative astigmatism and fast visual recovery. Accordingly, in this study, the vast majority of BCVA improvement was obtained at one month after surgery. Visual acuity slightly further improved at three-month follow-up probably due to the resolution of the three cases of cystoid macular edema and to corneal suture removal in patients who had removal of previously implanted dislocated IOL.

The anchor-shaped design of SSF IOL allowed in all cases a precise centration of the lens after haptic externalization without the need of further IOL manipulation by the surgeon. In case of IOL vertical tilt, IOL positioning was easily optimized by carefully rotating the anchors outside the sclerotomies. Furthermore, once externalized, the anchor of the leading haptic prevented posterior dislocation of the IOL, while the surgeon was fixating the trailing haptic that may occur with scleral glueing or scleral tucking techniques. Hence, implantation of SSF IOL allows a reduction of IOL fixation maneuvers in the vitreous cavity which may explain the absence of postoperative retinal break and/or detachment in our group. The accuracy of lens positioning was demonstrated by the low degree and the marked stability of horizontal and vertical tilt. Horizontal tilt was slightly greater than vertical tilt, probably due to mild asymmetry of the sclerotomies under the scleral flaps. None of the patients had a vertical or horizontal tilt exceeding 5°. Furthermore, no cases of IOL decentration or dislocation occurred during the whole follow-up. These results apparently compare favorably with those previously reported for sutured [25] and glued [26] scleral-fixated IOL. However, comparative studies are warranted to assess whether SSF IOL stability is superior to other techniques of IOL scleral fixation.

## 5. Conclusions

In conclusion, implantation of FILSSF is a safe and repeatable technique and provides good clinical outcomes with good visual outcomes, excellent IOL stability, and low complication rate. Limitations of this study include the relative low number of patients enrolled and short follow-up. Larger, long-term prospective studies are warranted to better assess the outcomes of this technique. Finally, prospective studies comparing FILSSF IOL with other scleral fixation techniques would be of interest.

## Data Availability

The data used to support the findings of this study are included within the article.

## References

[1] Popovic M., Campos-Möller X., Schlenker M. B., Ahmed I. I. K. (2016). Efficacy and safety of femtosecond laser-assisted cataract surgery compared with manual cataract surgery. *Ophthalmology*.

[2] Akkach S., Yip H., Meusemann R. (2019). Ten-year audit of posterior capsule tear complication rates and visual outcomes following phacoemulsification. *Clinical and Experimental Ophthalmology*.

[3] Por Y. M., Lavin M. J. (2005). Techniques of intraocular lens suspension in the absence of capsular/zonular support. *Survey of Ophthalmology*.

[4] Ascaso F. J., Huerva V., Grzybowski A. (2015). Epidemiology, etiology, and prevention of late IOL-capsular bag complex dislocation: review of the literature. *Journal of Ophthalmology*.

[5] Joshi R., Singanwad S. (2019). Frequency and surgical difficulties associated with pseudoexfoliation syndrome among Indian rural population scheduled for cataract surgery: hospital-based data. *Indian Journal of Ophthalmology*.

[6] Bergman M., Laatikainen L. (1997). Long-term evaluation of primary anterior chamber intraocular lens implantation in complicated cataract surgery. *International Ophthalmology*.

[7] Donaldson K. E., Gorscak J. J., Budenz D. L., Feuer W. J., Benz M. S., Forster R. K. (2005). Anterior chamber and sutured posterior chamber intraocular lenses in eyes with poor capsular support. *Journal of Cataract & Refractive Surgery*.

[8] Zeh W. G., Price F. W. (2000). Iris fixation of posterior chamber intraocular lenses. *Journal of Cataract & Refractive Surgery*.

[9] Lorencova V., Rozsival P., Urminsky J. (2007). Clinical results of the aphakia correction by means of secondary implantation of the iris fixated anterior chamber intraocular lens. *Ceská a Slovenská Oftalmologie*.

[10] Solomon K., Gussler J. R., Gussler C., Van Meter W. S. (1993). Incidence and management of complications of transsclerally sutured posterior chamber lenses. *Journal of Cataract & Refractive Surgery*.

[11] Asadi R., Kheirkhah A. (2008). Long-term results of scleral fixation of posterior chamber intraocular lenses in children. *Ophthalmology*.

[12] Apple D. J., Brems R. N., Park R. B. (1987). Anterior chamber lenses. Part I: complications and pathology and a review of designs. *Journal of Cataract & Refractive Surgery*.

[13] Patel S. R., Chu D. S., Ayres B. D., Hersh P. S. (2005). Corneal edema and penetrating keratoplasty after anterior chamber phakic intraocular lens implantation. *Journal of Cataract & Refractive Surgery*.

[14] Loya N., Lichter H., Goldenberg-Cohen N., Strassmann E., Weinberger D. (2001). Posterior chamber intraocular lens implantation after capsular tear: ultrasound biomicroscopy evaluation. *Journal of Cataract & Refractive Surgery*.

[15] Agarwal A., Kumar D. A., Jacob S., Baid C., Agarwal A., Srinivasan S. (2008). Fibrin glue-assisted sutureless posterior chamber intraocular lens implantation in eyes with deficient posterior capsules. *Journal of Cataract & Refractive Surgery*.

[16] Agarwal A., Kumar D. A., Jacob S., Prakash G., Agarwal A. (2009). Reply: fibrin glue-assisted sutureless scleral fixation. *Journal of Cataract & Refractive Surgery*.

[17] Kumar D. A., Agarwal A., Prakash G., Jacob S., Saravanan Y., Agarwal A. (2010). Glued posterior chamber IOL in eyes with deficient capsular support: a retrospective analysis of 1-year post-operative outcomes. *Eye*.

[18] Yamane S., Sato S., Maruyama-Inoue M., Kadonosono K. (2017). Flanged intrascleral intraocular lens fixation with double-needle technique. *Ophthalmology*.

[19] Kelkar A., Fogla R., Kelkar J., Kothari A., Mehta H., Amoaku W. (2017). Sutureless 27-gauge needle-assisted transconjunctival intrascleral intraocular lens fixation: initial experience. *Indian Journal of Ophthalmology*.

[20] Postorino M., Meduri A., Inferrera L. (2020). Scleral pockets for an innovative technique of intrascleral fixation of intraocular lens. *European Journal of Ophthalmology*.

[21] Kumar D. A., Agarwal A., Jacob S., Lamba M., Packialakshmi S., Meduri A. (2013). Combined surgical management of capsular and iris deficiency with glued intraocular lens technique. *Journal of Refractive Surgery*.

[22] Veronese C., Maiolo C., Armstrong G. W. (2020). New surgical approach for sutureless scleral fixation. *European Journal of Ophthalmology*.

[23] Rossi T., Iannetta D., Romano V. (2021). A novel intraocular lens designed for sutureless scleral fixation: surgical series. *Graefes Archive for Clinical and Experimental Ophthalmology*.

[24] Toro M. D., Longo A., Avitabile T. (2019). Five-year follow-up of secondary iris-claw intraocular lens implantation for the treatment of aphakia: anterior chamber versus retropupillary implantation. *PLoS One*.

[25] Marianelli B. F., Mendes T. S., de Almeida Manzano R. P., Garcia P. N., Teixeira I. C. (2019). Observational study of intraocular lens tilt in sutureless intrascleral fixation versus standard transscleral suture fixation determined by ultrasound biomicroscopy. *International Journal of Retina and Vitreous*.

[26] Kumar D. A., Agarwal D. A., Dhawan D. (2020). Glued intraocular lens in eyes with deficient capsules: a retrospective analysis of long term effects. *Journal of Cataract & Refractive Surgery*.

